# Tusanqi-Related Sinusoidal Obstruction Syndrome in China: A Systematic Review of the Literatures

**DOI:** 10.1097/MD.0000000000000942

**Published:** 2015-06-12

**Authors:** Xiaoxi Wang, Xingshun Qi, Xiaozhong Guo

**Affiliations:** From the Department of Gastroenterology, General, Hospital of Shenyang Military Area (XW, XQ, XG); and Postgraduate College, Liaoning University of Traditional Chinese Medicine (XW), Shenyang, China.

## Abstract

In West, sinusoidal obstruction syndrome (SOS) is often complicated with hemopoietic stem cell transplantation. By comparison, in China, SOS is frequently caused by Tusanqi-containing pyrrolizidine alkaloids. A systematic review aimed to evaluate the clinical profiles, diagnostic workup, treatment, and outcomes of Tusanqi-related SOS in China.

All relevant articles were searched via PubMed, China Knowledge Resource Integrated, VIP, and Wanfang databases. Case reports were defined, as the data were available in every individual patient. Otherwise, case series were defined.

Overall, 106 articles were eligible. Fifty-six case reports included 84 individual patients with SOS secondary to Tusanqi alone. All of them presented with ascites, but only 1 patient presented with upper gastrointestinal bleeding. The 1-, 3-, and 6-month cumulative survival rate was 98%, 87%, and 76%, respectively. Increased bilirubin and aspartate transaminase levels were significantly associated with poor outcome. Thirty-one case series included 402 patients with SOS secondary to Tusanqi alone. Ascites was observed in 94% of patients, but upper gastrointestinal bleeding was observed in 40% of patients. Recovery, stabilization, progression, and death were observed in 41%, 30%, 14%, and 16% of patients, respectively. Nineteen case series included 281 patients with SOS secondary to mixed etiologies. The pooled proportion of Tusanqi-related SOS was 66% (95% confidence interval: 56%–75%).

Tusanqi is a major cause of SOS in China. Ascites is the most common clinical presentation of Tusanqi-related SOS. Despite a relatively good short-term outcome, further studies should be necessary to explore the long-term outcome and refine the treatment strategy.

## Abstract 2

Supplemental Digital Content is available in the text

## INTRODUCTION

Sinusoidal obstruction syndrome (SOS) is a rare vascular disease of the liver, which can lead to lethal liver failure and portal hypertension-related complications.^1^ SOS is formerly called as veno-occlusive disease. The change in the nomenclature of this disease is primarily because the major lesion is located at the hepatic sinusoids.^2^ The classical triads of SOS include ascites, hepatomegaly, and increased bilirubin levels. In the West, the most common cause of SOS is the conditioning treatment with chemotherapy before hemopoietic stem cell transplantation (HSCT).^3,4^ SOS can develop within approximately 20 to 30 days of HSCT.^5,6^ By comparison, in China, SOS is usually caused by herbal medicine containing pyrrolizidine alkaloids.^7^ The most frequent herbal medicine reported is Tusanqi (ie, gynura segetum), which is used to relieve pain, improve blood circulation, and dissipate blood stasis. Until now, Tusanqi-related SOS has been reported by scattered cases.^8–10^

Given the difference in the cause of SOS between the West and China, the clinical characteristics and outcomes of Western cases with SOS might be hardly extrapolated to Chinese cases. Herein, we tried our best to collect all relevant literatures to systematically evaluate the clinical profiles, diagnostic workup, treatment, and outcomes of Tusanqi-related SOS in China.

## METHODS

This systematic review was registered with PROSPERO (registration number: CRD42015019163).

### Search Strategy

All relevant articles were searched via PubMed, China Knowledge Resource Integrated, VIP, and Wanfang databases. The first one is a major English-language database, and the latter 3 are Chinese-language databases. English-language search items were used in PubMed database, as follows: (((sanqi) *OR* pyrrolizidine alkaloids)) AND ((sinusoidal obstruction syndrome) *OR* hepatic venoocclusive disease). Chinese-language search items were used in latter 3 databases, as follows: ((san qi) *OR* (bi luo wan)) AND ((gan dou zu se) *OR* (gan xiao jing mai bi se)). Last search was performed on March 26, 2015.

### Study Selection

All clinical studies were considered, if they reported the cases with Tusanqi-related SOS. Exclusion criteria were as follows:

1. Duplicates among databases;
2. Duplicate publications by the same author in different journals;
3. Reviews;
4. Comments or editorials;
5. Newspapers;
6. Indexes;
7. Basic studies;
8. Irrelevant articles;
9. Lacking of detailed data;
10. Overlapping data.

Notably, if SOS was not secondary to Tusanqi, but other drugs, the articles would be excluded. In addition, we rechecked the diagnostic workup of the original articles to avoid the misdiagnosis of SOS. If a diagnosis of SOS was unclear, the cases would be excluded. We also rechecked all cases with the same age, sex, hospitalization, and date of admission to avoid the inclusion of overlapping data. If the data were overlapping among 2 or more articles, only 1 article with more adequate baseline and follow-up data or a longer enrollment period would be included.

### Data Extraction

According to the ways of data expression, all included studies were divided into case reports and case series. Case reports were defined, if we could extract the clinical data of every individual patient with Tusanqi-related SOS. Otherwise, case series were defined. Furthermore, they were classified as 2 types according to the proportion of Tusanqi-related SOS. Type I: all observed patients developed SOS secondary to Tusanqi. Type II: only a proportion of patients developed SOS secondary to Tusanqi.

We extracted the following data: sex, age, indication, form, dosage, frequency, and duration of Tusanqi, clinical presentation and physical sign, gastroesophageal varices, diagnostic method for SOS, treatment, and outcome. Survival time was defined as the interval between the first admission and death or last follow-up. Notably, we also extracted the proportion of Tusanqi-related SOS from type II case series.

### Study Quality

Given the nature of included studies (ie, case reports and noncomparative case series), no preexisting quality assessment scale was well established. We developed the following items to evaluate the study quality and data completeness.

1. Patient enrollment: are the patients consecutively and prospectively enrolled?
2. Demographic data: are the sex and age clearly reported?
3. Clinical presentations: are the clinical presentations clearly reported?
4. Diagnosis: is the diagnostic workup clearly reported?
5. Management: is the treatment clearly reported?
6. Outcome: is the outcome clearly reported?

### Data Analysis

As for the case reports, the statistical analyses were performed by SPSS Statistics software version 17.0.0 (Chicago, Illinois, USA). The continuous data were expressed as mean ± standard deviations and/or median (range), and categorical data were expressed as frequency (percentage). Based on the survival time and status available from these individual cases, we drew Kaplan–Meier curves. Cumulative survival rates were reported. In addition, Cox regression analysis was performed to evaluate the factors that were significantly associated with survival. Hazard ratios (HRs) with 95% confidence intervals (CIs) for every variable were calculated. The factors that were statistically significant in univariate analysis were included in the multivariate analysis. A *P* value <0.05 was considered as statistically significant.

As for the case series, the meta-analyses were performed by StatsDirect Statistical Package software version 2.7.8 (StatsDirect Ltd, Sale, Cheshire, UK). The proportion of major clinical profiles in patients with Tusanqi-related SOS was extracted from type I case series, and then they were combined into a general proportion. Similarly, the proportion of Tusanqi-related SOS in all patients with SOS secondary to mixed etiologies was extracted from type II case series, and then they were combined into a general proportion. A random-effects model was used to perform a meta-analysis. Heterogeneity among studies was calculated. *I*^2^ > 50% or *P* < 0.1 was considered to have a statistically significant heterogeneity.

## RESULTS

### Study Selection

**FIGURE 1:**
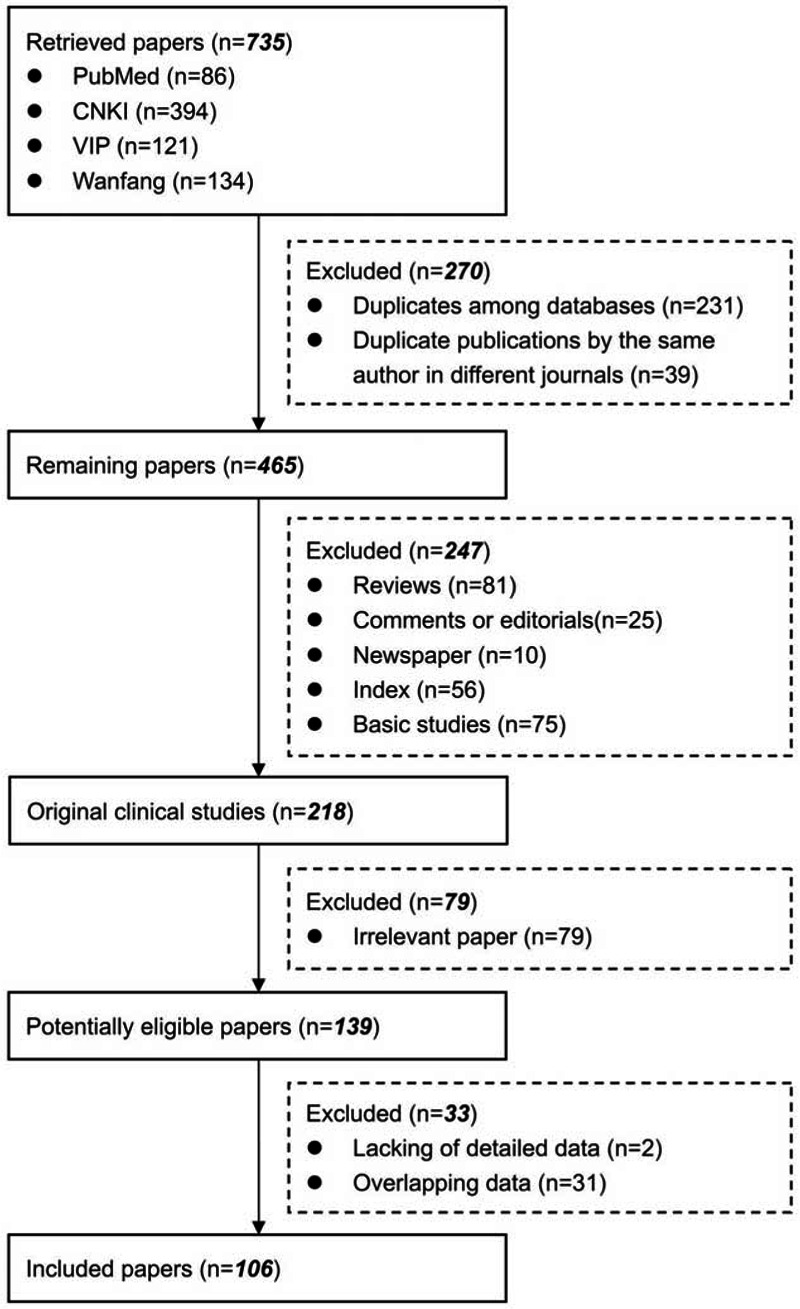
Flowchart of study inclusion.

Overall, 735 articles were retrieved. Among them, 139 articles were in potentially eligible in the systematic review (Figure 1). Two articles that were lacking of detailed data were excluded. Another 31 articles with overlapping data were also excluded (Supplementary Table 1, http://links.lww.com/MD/A297). Thus, 106 articles were finally included. They included 56 case reports and 50 case series.

### Case Reports

Information of 56 case reports was summarized in Supplementary Table 2, http://links.lww.com/MD/A297. They included 84 individual cases with Tusanqi-related SOS from 49 affiliations in 19 provinces or regions. These patients were admitted between 1999 and 2013.

Patient enrollment was not evaluated in any case reports. The demographic data, clinical profiles, and diagnostic workup were clearly available in all of 84 individual cases. The treatment and outcome were clearly available in 73 individual cases (Supplementary Table 3, http://links.lww.com/MD/A297).

Patient characteristics were summarized in Table 1. Ascites was the most common clinical presentation at their admission. By contrast, only 1 of them presented with upper gastrointestinal bleeding.

**TABLE 1:**
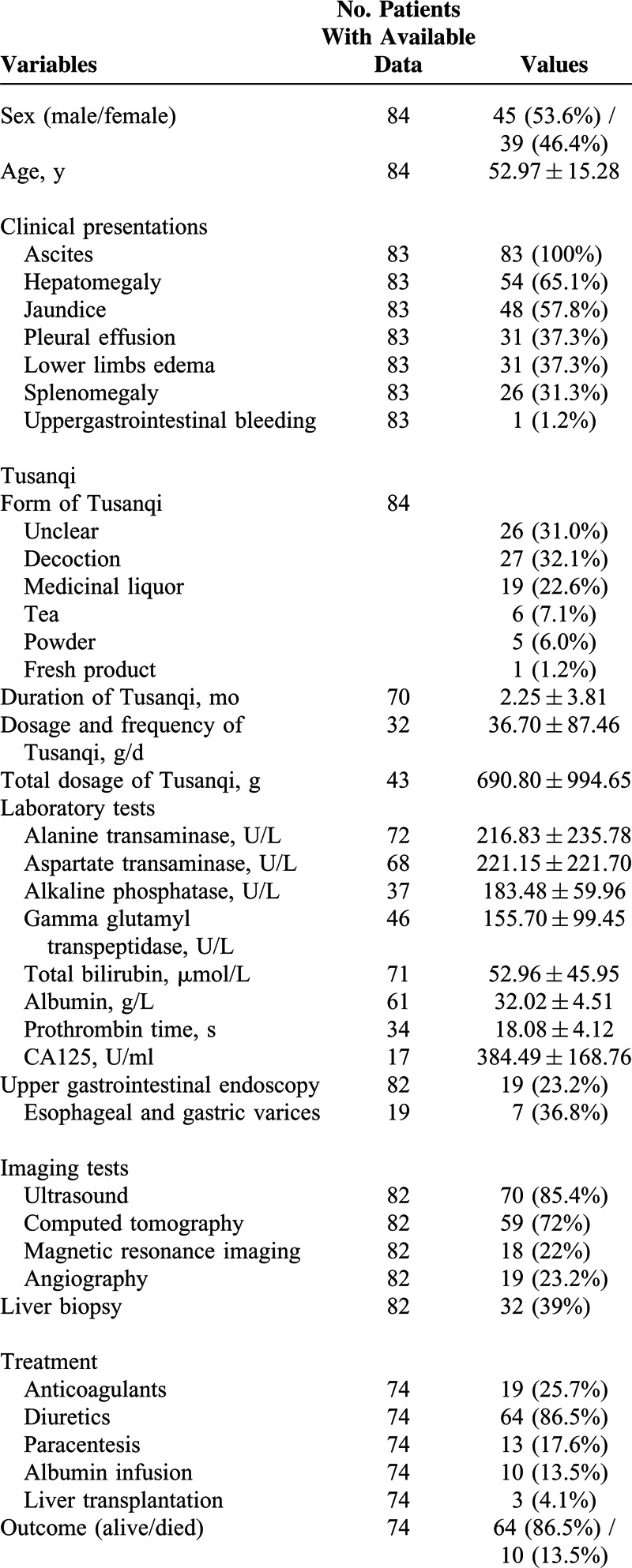
Characteristics of 84 Cases From Case Reports

Decoction was the most common form of Tusanqi. The average duration between the use of Tusanqi and first onset of clinical symptoms was 2.25 ± 3.81 months. The median duration was 1 month (range: 0.2–24). The average total dosage of Tusanqi was 690.80 ± 994.65 g. The median total dosage was 450 g (range: 10–5000).

Nineteen patients underwent upper gastrointestinal endoscopy, of whom 7 (39%) had positive gastroesophageal varices. Most of patients underwent ultrasound and computed tomography for the diagnosis of SOS. However, only 32 patients underwent liver biopsy.

Conventional therapy was prescribed for the treatment of ascites in a majority of patients, including diuretics (n = 64), paracentesis (n = 13), and albumin infusion (n = 10). Anticoagulation therapy was prescribed in 19 patients. Liver transplantation was performed in only 3 patients.

**FIGURE 2:**
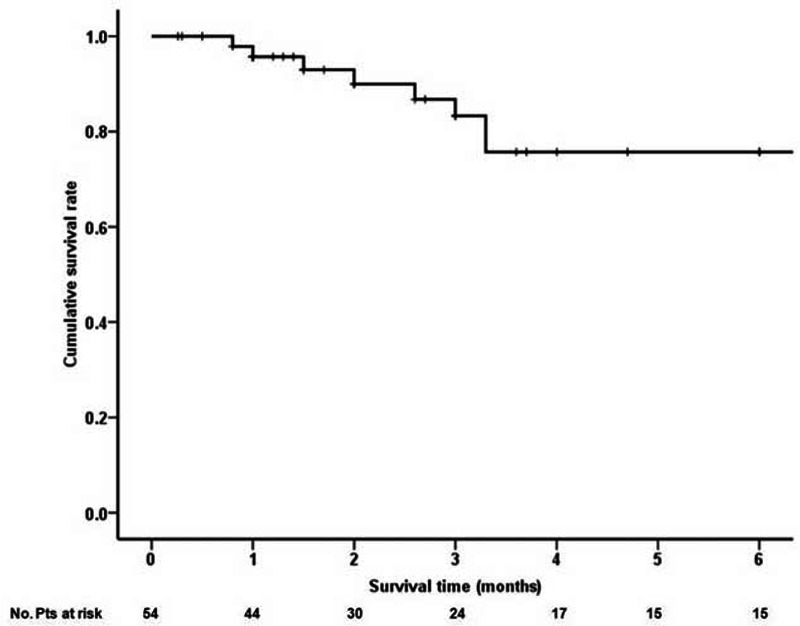
Kaplan–Meier curve showing the cumulative survival of 54 individual cases.

The survival time and status were available in 54 individual cases. The 1-, 2-, 3-, and 6-month cumulative survival rate was 97.9%, 93%, 86.8%, and 75.7%, respectively (Figure 2). In the univariate Cox regression analysis, only increased total bilirubin levels (HR = 1.014, 95% CI = 1.005–1.023, *P* = 0.003) and aspartate transaminase levels (HR = 1.003, 95% CI = 1.001–1.005, *P* = 0.012) were significantly associated with poor survival (Table 2). In the multivariate analysis, increased total bilirubin levels (HR = 1.024, 95% CI = 1.003–1.045, *P* = 0.027) and aspartate transaminase levels (HR = 1.004, 95% CI = 1.001–1.007, *P* = 0.005) remained significantly associated with poor survival.

**TABLE 2:**
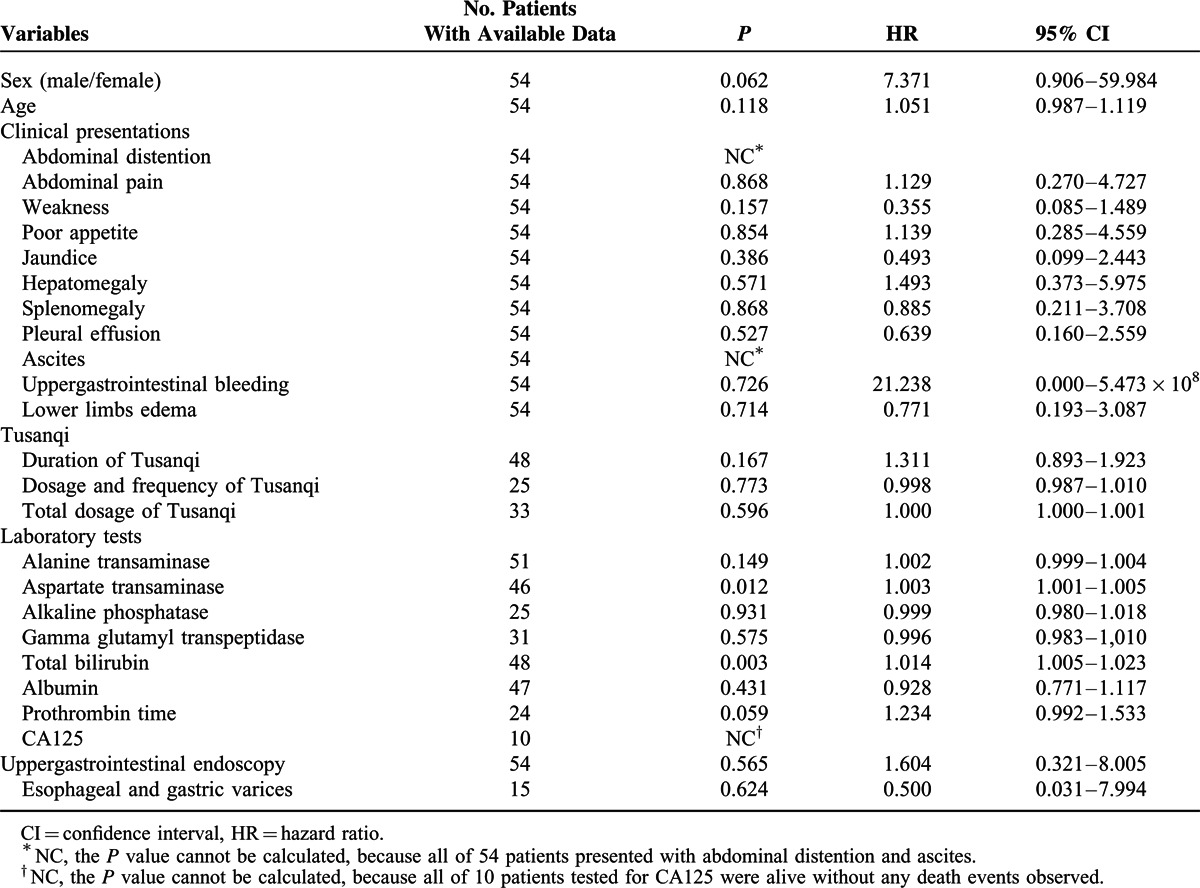
Prognostic Factors in 54 Patients From Case Reports

### Type I Case Series

Information of 31 type I case series was summarized in Supplementary Table 4, http://links.lww.com/MD/A297. They included 402 individual cases with Tusanqi-related SOS from 29 affiliations in 11 provinces or regions. These patients were admitted between 1996 and 2014.

Patient enrollment was neither consecutive nor prospective in any case series. The demographic data were clearly available in 30 of 31 case series. The clinical profiles and diagnostic workup were clearly available in all case series. The treatment was clearly available in 21 of 31 case series. The outcome was clearly available in 20 of 31 case series (Supplementary Table 5, http://links.lww.com/MD/A297).

Patient characteristics were summarized in Table 3   . The most common clinical profile was ascites (94.0%, 95% CI = 88.5%–97.8%) (Supplementary Figure 1, http://links.lww.com/MD/A297), followed by hepatomegaly (88.7%, 95% CI = 80.5%–94.8%) (Supplementary Figure 2, http://links.lww.com/MD/A297), jaundice (70.4%, 95% CI = 58.0%–81.5%) (Supplementary Figure 3, http://links.lww.com/MD/A297), plueral effusion (64.1%, 95% CI = 20.7%–96.6%) (Supplementary Figure 4, http://links.lww.com/MD/A297), lower limbs edema (46.5%, 95% CI = 25.8%–67.9%) (Supplementary Figure 5, http://links.lww.com/MD/A297), splenomegaly (40.8%, 95% CI = 20.9%–62.5%) (Supplementary Figure 6, http://links.lww.com/MD/A297), uppergastrointestinal bleeding (40.3%, 95% CI = 11.3%–73.6%) (Supplementary Figure 7, http://links.lww.com/MD/A297), gastroesophageal varices (35.4%, 95% CI = 11.2%–64.5%) (Supplementary Figure 8, http://links.lww.com/MD/A297), and hepatic encephalopathy (17.6%, 95% CI = 10.9%–25.5%) (Supplementary Figure 9, http://links.lww.com/MD/A297). In the meta-analysis regarding hepatic encephalopathy, the heterogeneity was not statistically significant among studies (*I*^2^ = 0%, *P* = 0.9328). But in the other meta-analyses, the heterogeneity was statistically significant among studies.

**TABLE 3:**
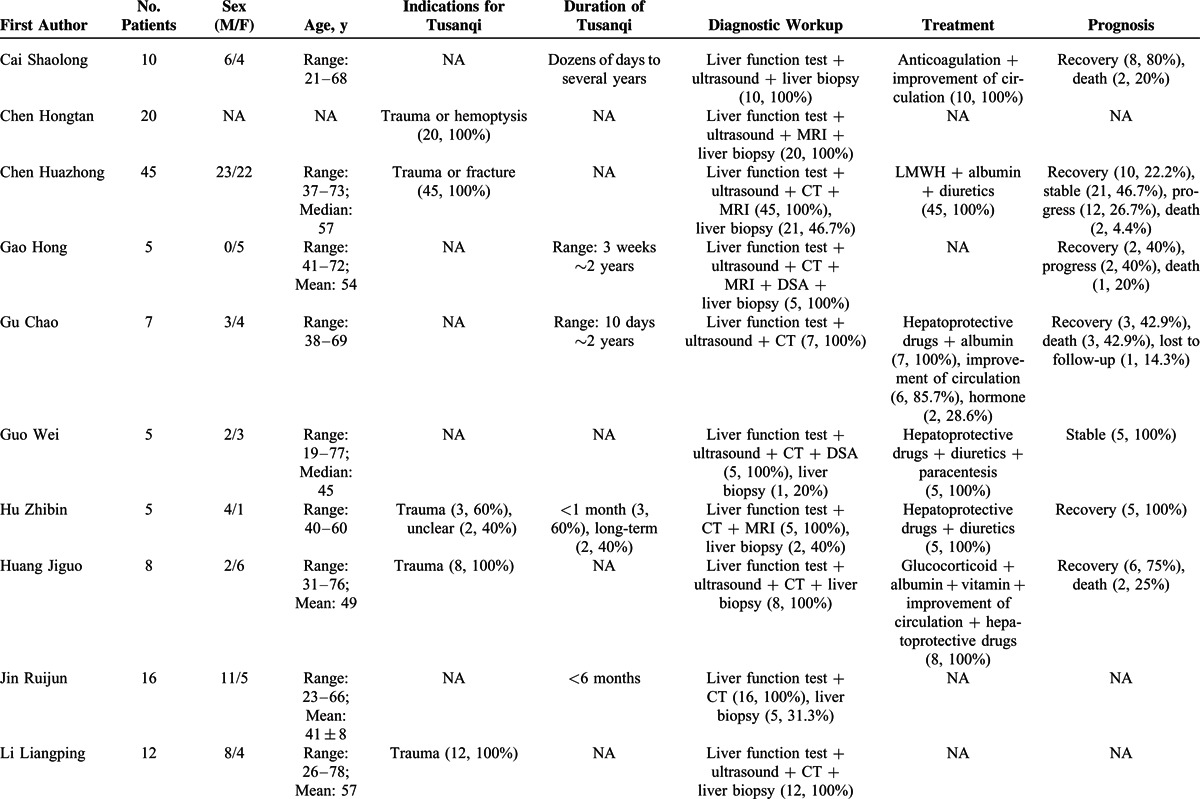
Patient Characteristics of 31 Case Series (Type 1)

**TABLE 3 (Continued):**
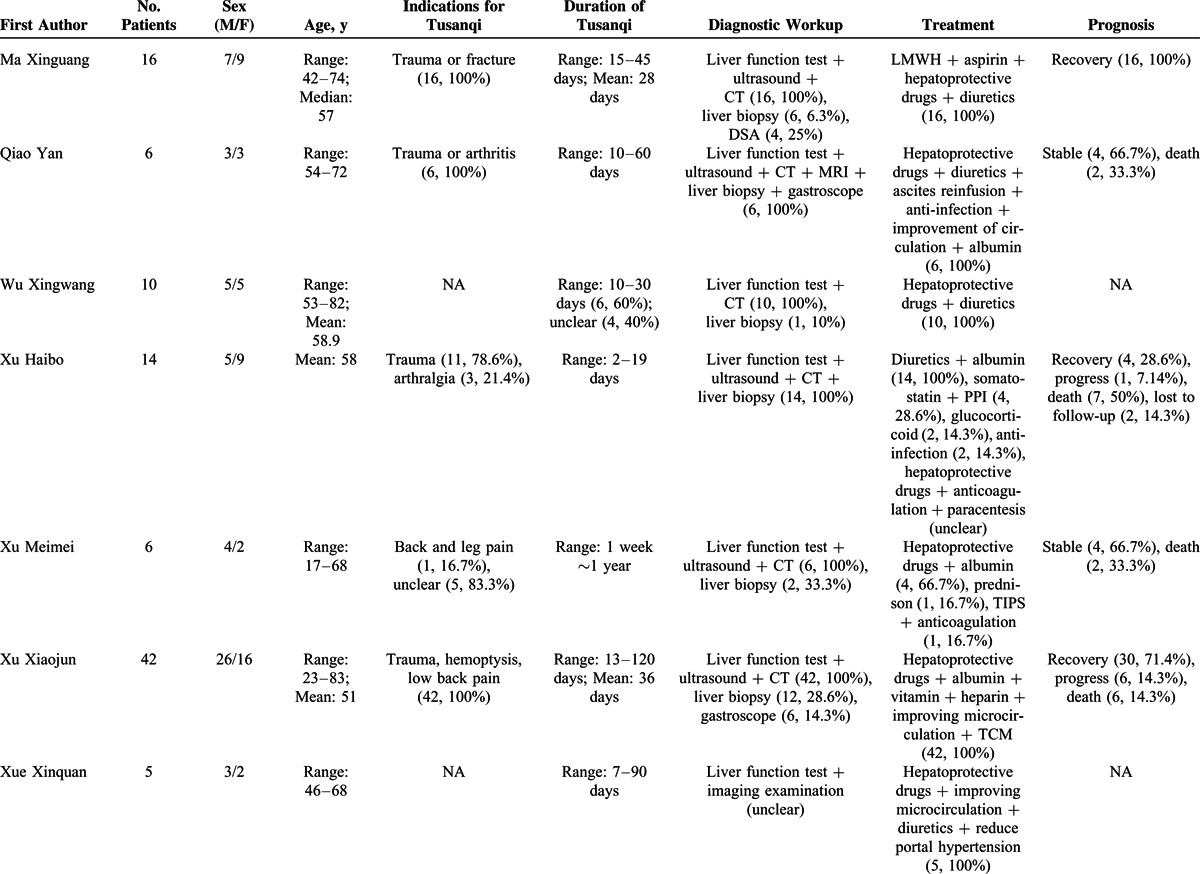
Patient Characteristics of 31 Case Series (Type 1)

**TABLE 3 (Continued):**
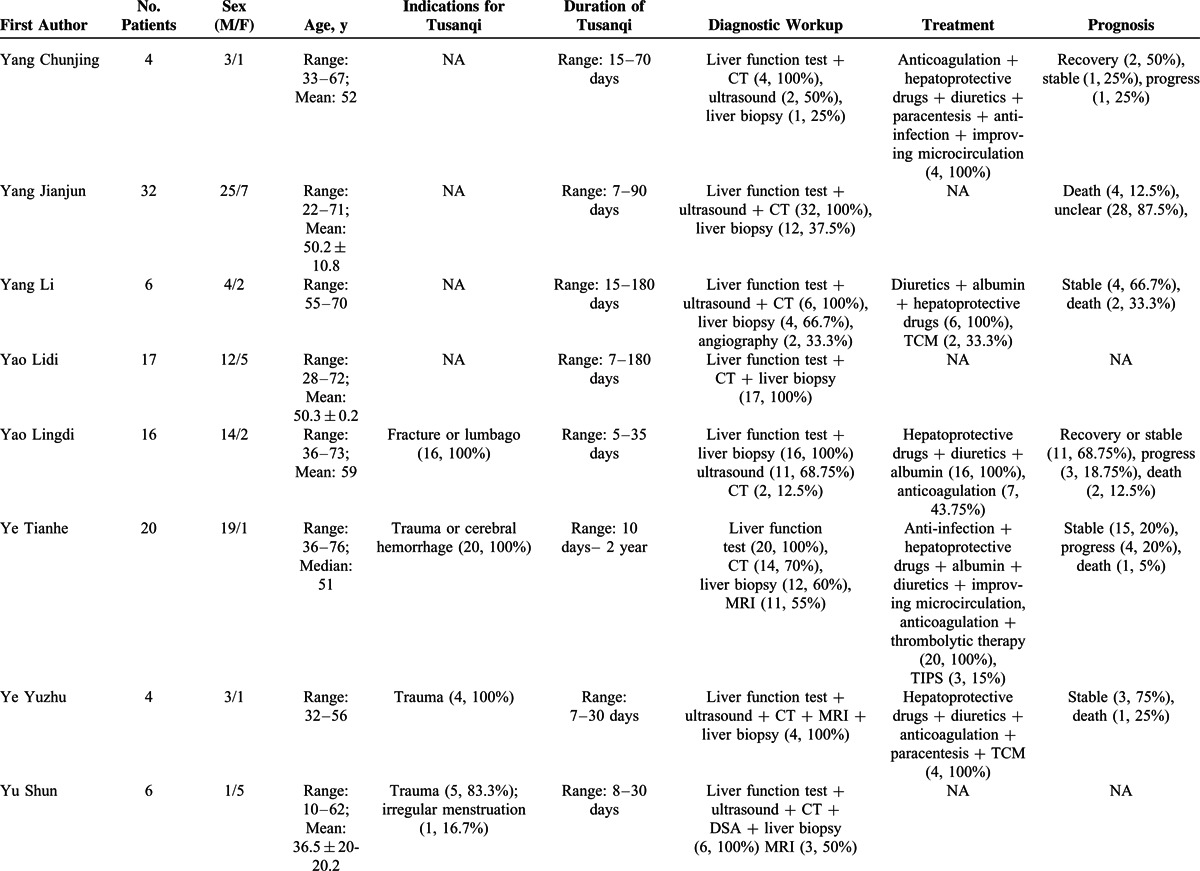
Patient Characteristics of 31 Case Series (Type 1)

**TABLE 3 (Continued):**
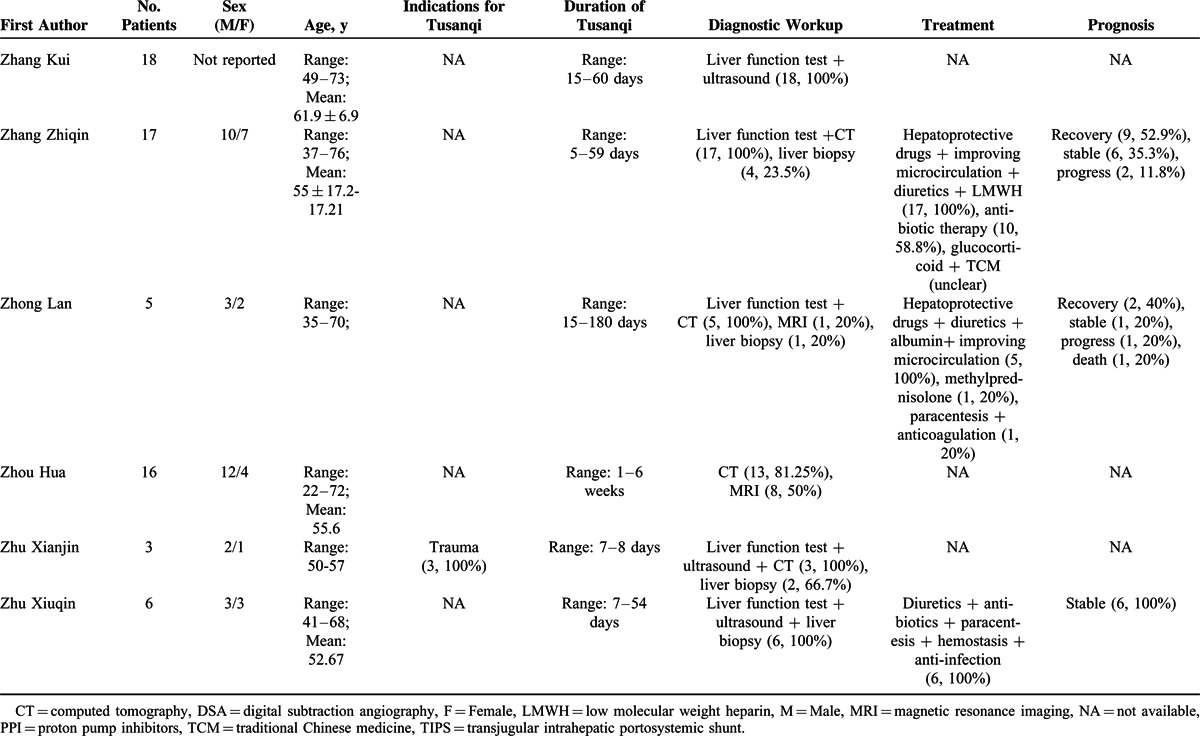
Patient Characteristics of 31 Case Series (Type 1)

The indications for Tusanqi were clearly reported in 15 cases series. They included trauma, arthritis, arthralgia, back pain, leg pain, hemoptysis, cerebral hemorrhage, and irregular menstruation. The forms of Tusanqi were clearly reported in 16 case series. They included decoction, liquor, powder, tea, and steam. The duration between the use of Tusanqi and first onset of clinical symptoms was reported in 26 case series. The duration was heterogeneous, ranging from 2 days to 2 years. The total dosage of Tusanqi was clearly reported in 11 case series. The dosage was heterogeneous, ranging from 100 to 1500 g.

Diagnostic workup was clearly reported in all case series. Liver biopsy was used in 210 patients from 27 case series.

Treatment was reported in 21 case series. Anticoagulation was used in 167 patients from 12 case series. Transjugular intrahepatic portosystemic shunt (TIPS) was used in 4 patients from 2 case series. Liver transplantation was not used in any patients.

Outcome was reported in 237 patients from 21 case series. They included recovery in 97 (40.9%) patients from 13 case series, stabilization in 70 (29.5%) patients from 12 case series, progression in 32 (13.5%) patients from 9 case series, and death in 38 (16.1%) patients from 15 case series.

### Type II Case Series

Information of 19 type II case series was summarized in Supplementary Table 6, http://links.lww.com/MD/A297. They included 281 cases with SOS secondary to mixed etiologies from 18 affiliations in 6 provinces or regions. These patients were admitted between 2003 and 2014.

Patient enrollment was neither consecutive nor prospective in any case series. The demographic data was clearly available in 17 of 19 case series. The clinical profiles were clearly available in 15 of 19 case series. The diagnostic work-up was clearly available in 17 of 19 case series. The treatment was clearly available in 13 of 19 case series. The outcome was clearly available in 12 of 19 case series (Supplementary Table 7, http://links.lww.com/MD/A297).

**FIGURE 3:**
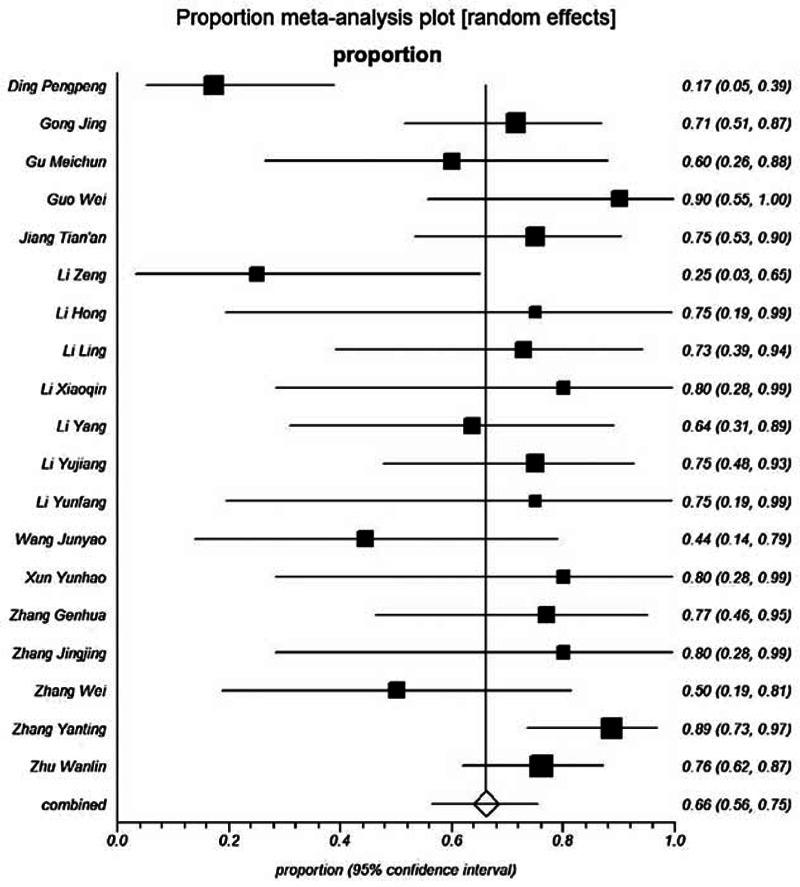
Forest plot showing the proportion of Tusanqi-related SOS.

The proportion of Tusanqi-related SOS ranged from 17.4% to 90%. The pooled proportion was 66.2% (95% CI = 56.4%–75.3%) (Figure 3). The heterogeneity was statistically significant among studies (*I*^2^ = 64.3%, *P* < 0.0001).

## DISCUSSION

This article systematically reviewed the currently available literatures to evaluate the clinical characteristics of Tusanqi-related SOS in China. Several strengths should be clarified. First, the search strategy was extensive via 4 major databases. We maximized the number of relevant articles by searching “sanqi” and “pyrrolizidine alkaloids,” but not “Tusanqi” alone. Overall, a relatively large number of eligible articles were identified (n = 106). Among them, 56 case reports included 84 individual cases with Tusanqi-related SOS, and 31 type I case series included 402 individual cases with Tusanqi-related SOS. Second, as we previously described, the case of duplicate publication was frequently observed in the Chinese literatures.^11^ To avoid the inflation of relevant data, we carefully excluded all duplicate articles according to the same affiliations and overlapped enrollment periods. All detailed information regarding the overlapping data was clearly shown in Supplementary Table 1, http://links.lww.com/MD/A297). Third, because Tusanqi-related SOS is rarely observed at a single center, a small number of cases greatly precludes from the prognostic assessment of Tusanqi-related SOS. By comparison, we systematically collected all individual data from case reports. Cox regression analysis became feasible in 54 cases with survival data. Fourth, meta-analyses were also used to combine the proportion of Tusanqi-related SOS in different studies.

Our study found that most of patients with Tusanqi-related SOS were admitted due to ascites and hepatomegaly; by comparison, only a minority of patients presented with uppergastrointestinal bleeding at their admission. This might be attributed to a relatively short follow-up duration, which restricted our observations of the transition from acute to chronic stage. These findings suggest that previous history of herbal medicine should be carefully inquired, if the reasons for ascites were ambiguous.

In West, SOS is often secondary to HSCT. The pathogenesis of SOS during HSCT is hypothesized as the activation of the hepatic sinusoidal endothelial cells by various factors and the subsequent toxic destruction of endothelial cells with sloughing and downstream occlusion of terminal hepatic venules.^4^ Contributing factors include glutathione and nitric oxide depletion of sinusoidal endothelial cells, increased intrahepatic expression of matrix metalloproteinases and vascular endothelial growth factor, and activation of coagulation cascade. However, whether or not these mechanisms could be extrapolated to the Tusanqi-related SOS remained uncertain.

Recently, in an animal study, Fang et al^12^ observed the hematologic toxicity of Tusanqi and its effects on vascular endothelium. Compared with control group, the rats with Tusanqi-related SOS had a decrease in platelets and platelet hematocrit and an increase in mean platelet volume and platelet distribution. This seemed to be consistent with our findings that 30% to 40% of patients with Tusanqi-related SOS presented with splenomegaly. Indeed, the multivariate analysis by Imai et al. also found that the increase in the splenic volume should be an independent predictor of the development of SOS in patients who received oxaliplatin-based chemotherapy with or without bevacizumab for colorectal liver metastases.^13^ These findings suggested the role of splenomegaly in the early diagnosis and pathogenesis of SOS.

Furthermore, the animal study by Fang et al^12^ demonstrated that the serum levels of endothelin and nitric oxide were elevated and the histology of spleen was disrupted (ie, the splenic follicles were significantly reduced, and the germinal centers disappeared in the spleen, and the red pulp sinusoids were reduced). Given the cross-talk between endothelium and lymphocyte network and the intersection of liver–spleen axis among many aspects,^14,15^ it may be more likely that splenomegaly in Tusanqi-related SOS is caused by the change in endothelin, but not necessarily linked to chronic portal hypertension. Indeed, our study also demonstrated that variceal bleeding secondary to portal hypertension was less frequent than splenomegaly.

Another finding was that some patients could rapidly develop SOS-induced symptoms within several days. Based on the statistical analysis of case reports, the average duration from the use of Tusanqi to the first onset of clinical symptoms was about 2 months, and the median duration was about 1 month. These findings suggested that hepatic sinusoidal injury could be immediately caused by Tusanqi. Certainly, the duration was widely heterogeneous among studies. The duration might be influenced by the total dosage, frequency, and form of Tusanqi. In future, animal studies were valuable to establish their association and determine the maximal tolerable dosage and frequency of Tusanqi.

In the West, the currently well-established diagnostic criteria for SOS include original or modified Seattle criteria and Baltimore criteria.^16–19^ Bilirubin, hepatomegaly, ascites, and weight gain are the 4 major components of these criteria. Except for the major diagnostic criteria, our systematic review also disclosed that nearly half of patients with Tusanqi-related SOS underwent liver biopsy.

The symptomatic treatments were used in most of patients with Tusanqi-related SOS, including hepatoprotective drugs, diuretics, paracentesis, and albumin infusion. In addition, anticoagulation therapy, TIPS, and liver transplantations were used in few patients. However, we could not accurately evaluate the safety and efficacy of these treatment modalities according to the limited data.

Patients with Tusanqi-related SOS had a relatively good outcome. Most of death events occurred during a relatively short follow-up period. However, the long-term follow-up was lacking. In addition, the most common cause of death might be acute liver failure, rather than portal hypertension-related complications. Cox regression analysis also found that increased total bilirubin and aspartate transaminase levels, the 2 major variables reflecting the severity of liver dysfunction, were significantly associated with poor outcome. Certainly, it should be noted that the HRs for the 2 variables were very close to 1. Their prognostic values should be further confirmed.

This study had several limitations. First, the quality of included studies was relatively poor. A majority of studies were case reports. Due to the rarity of this disease, the patient enrollment might be retrospective in most of case series. Second, only a short-term follow-up result could be provided in this systematic review. Third, although our meta-analysis found that Tusanqi might be the most common cause of SOS in China (66.2%), we should never neglect the heterogeneity among studies. In addition, we had to acknowledge the potential clinical benefits of this herbal remedy. However, we cannot obtain the information regarding the incidence of Tusanqi-related SOS in all persons receiving Tusanqi. Thus, the adverse effects of Tusanqi should not be overestimated based on our findings. Finally, clinical characteristics and outcomes could not be compared between patients with Tusanqi-related SOS and those with SOS secondary to other etiologies.

In conclusions, our study demonstrated the following: Tusanqi was a major cause of SOS in China; ascites and hepatomegaly were 2 most common clinical profiles of Tusanqi-related SOS; SOS could develop within several days after the use of Tusanqi; the short-term outcome was relatively good; and increased bilirubin and aspartate transaminase levels were significantly associated with poor outcomes. These preliminary findings were important for physicians and patients to recognize the Tusanqi-related SOS in China. Further studies should be necessary to explore the long-term follow-up results, refine the treatment strategy of Tusanqi-related SOS, and evaluate the indications of different treatment options.
